# Pituitary Tumors: New Insights into Molecular Features, Diagnosis and Therapeutic Targeting

**DOI:** 10.3390/cancers13071697

**Published:** 2021-04-03

**Authors:** Monica Fedele

**Affiliations:** National Research Council (CNR), Institute of Experimental Endocrinology and Oncology (IEOS), 80145 Naples, Italy; mfedele@unina.it

In this Special Issue, a series of eight original research articles and six reviews have been collected to highlight the latest knowledge into molecular features, diagnosis and therapeutic targeting of pituitary tumors, one of the most frequent intracranial tumors and neuroendocrine neoplasms. Pituitary tumorigenesis is driven by various mechanisms, the knowledge of which could be useful for the development of effective therapeutic strategies, even for the most aggressive subtypes, characterized by invasiveness, high proliferative index, relapse and resistance to conventional treatment. We are particularly pleased to have reports in this series that focus on aggressive pituitary tumors that most urgently need new therapeutic approaches.

The Special Issue starts with Trouillas et al., discussing the current WHO 2017 classification of these tumors and introducing an alternative five-tiered prognostic classification, which takes into account invasion, immuno-histochemical (IHC) type and proliferative markers (Ki-67 index, mitotic count, p53 positivity) [1]. On the same topic, Picò’s group shows that the molecular determination of the pituitary-specific transcription factors and hormone genes would significantly integrate the IHC studies for the identification of pituitary neuroendocrine tumor subtypes according to the WHO 2017 criteria [2].

Serioli et al. present a complete and detailed review about invasiveness of pituitary adenoma [3], while Lamb et al. review previous and current studies regarding therapies for aggressive pituitary tumors, discussing the appropriateness and effectiveness role of novel medical therapies [4]. In this frame, the original research by Vázquez-Borrego et al. provides evidence that spliceosome machinery is dysregulated in a tumor subtype-dependent manner, being associated with aggressive features, suggesting the use of specific splicing-machinery components as novel diagnostic/prognostic and therapeutic targets in these tumors [5]. On the other hand, Fusco’s group represent the state of the art on the emerging role of USP8, HMGA proteins and Non-coding RNA in pituitary tumorigenesis [6], while Shah and Aghi provide a narrative review of the current literature on single nucleotide polymorphisms in human pituitary adenomas [7]. The tumor microenvironment of pituitary tumors is the protagonist of the very interesting and timely review by Ilie et al. [8], which deals with the different aspects of this extracellular component in pituitary tumor pathogenesis and in targeted therapy, suggesting further studies in this direction to provide effective and personalized treatment for patients.

The series continues with an original research article on an innovative diagnostic method, based on metabolite composition of surgically resected Rathke’s Cleft Cyst (RCC), by Ijare et al. [9]. The authors show metabolites, including N-acetyl sugars from mucopolysaccharides and glycosaminoglycans, which are not detected in both functional and non-functional pituitary adenomas, opening important perspectives in the differentiation of RCC from pituitary adenomas. The gender difference of pituitary adenomas is uniquely addressed in the paper by Pecori Giraldi et al. [10], which shows for the first time that human adrenocorticotropic hormone (ACTH)-producing adenomas exhibit sexual dimorphism and stresses the need for a gender-dependent analysis of these tumors. Cushing’s disease, the human condition caused by ACTH-producing adenomas of the pituitary, is the focus of the research article by Sbiera et al. [11], who performed a meta-analysis of genetic data related to nine studies, revealing USP8 and USP48 mutations as the most frequent driver genes in this disease. Another pituitary adenoma histotype, the gonadotroph nonfunctioning pituitary tumor (NFPA), is the object of the research by Bujko’s group [12], which presents a detailed analysis of CpGs methylation profile in NFPAs in reference to gene expression, determining the ratio of differentially expressed genes and tumor-related pathways that may be regulated by DNA methylation. The last two articles present long-term follow up studies. On the one hand, Chang et al. analyzed data from 248 patients with NFPA macroadenomas, with the aim of comparing toxicities among three treatment groups, including contemporary stereotactic radiosurgery, fractioned radiotherapy and transphenoidal surgery [13]. On the other hand, Kessel et al. retrospectively analyzed 69 patients with secreting and non-secreting pituitary tumors over a 17-year follow up to compare the outcome after fractionated stereotactic radiotherapy and the patient-reported outcomes [14].

In conclusion, this Special Issue provides a well-rounded view of the latest findings on pituitary tumors, starting with their classification and differential diagnosis, which includes the identification of new potential biomarkers suitable for diagnosis and future targeted therapies, up to studies on patients to analyze the toxicity and patient outcome of different surgical or radiotherapy treatment options. From this overview, it emerges that new pituitary tumor biomarkers are needed and that they represent a very varied and complex research field. Their discovery is not only important for the diagnostics, but will open up new therapeutic options especially needed for aggressive pituitary tumors.
